# *Reports on Cities, Towns, and Districts*.—Huddersfield

**Published:** 1857-04

**Authors:** 


### SANITARY AND SOCIAL SCIENCE.

### REPORTS ON CITIES, TOWNS, & DISTRICTS.

### THE SANITARY CONDITION OF HUDDERSFIELD.

HUDDERSFIELD, one of the principal seats of the woollen manufacture, is situated in the West Riding of Yorkshire, 24 miles north-east of Manchester, and 189 miles north-north-west of London. This town may be considered as the centre of a highly populous and flourishing neighbourhood, comprising as it does within a radius of six miles in circumference, a population considerably exceeding 100,000 persons, and being located within easy distances of the great towns of Leeds, Bradford, Halifax, Sheffield, and Wakefield. The town itself stands on a plain, on the banks of the river Colne, for the most part declining from the north-west to the east, but also declining to the river on the south, and to the Hill House Valley on the north; these large slopes afford great natural facilities for removing the town's drainage. The height above the sea, at the river to the east, is 200 feet; to the north-west, near Edgerton, 446 feet; to the south, at Longroyd Bridge, on the Colne, 260 feet; and to the Hill House Valley, near Bayhall, 250 feet. Castle Hill frowns over the town at a short distance to the south, at a height of 900 feet above the sea level; to the north-east, the Whitley Hall and Grange Moor range of hills attain the height of 775 feet; the intervening hills of Almondbury Bank and Kilner Bank are respectively, at their summit level, the former 500 feet, the latter 400 feet; to the north-by-west of the town, the Ainley and Fixby ridge varies from 693 feet to 850 feet above the sea level, and this range of hills is remarkable for its treasure of the finest grey slates and flag stones; whilst to the south-west, the altitude of the hills varies from 600 to 1,000 feet; these elevated ranges form a part of that remarkable chain of mountains, called the back bone of Yorkshire, situate between Blackstone Edge and Standage.

GEOLOGICAL FEATURES. It cannot fail to strike the beholder that our town is situated where once great convulsions have taken place. We have evidence of this in the strata upon which the town is chiefly built; for, after removing the alluvial soil on the surface, we find beds of sand and clay containing large pieces of argillaceous sandstone, brought from a distance, sometimes in groups and sometimes disseminated amongst the clay, some of them evidently waterworn. We also meet with boulders of a rock, locally termed "Ganister," also brought from a distance and waterworn; abundance of them are found at Hill House, buried in the clay: they are of great specific gravity, and contain beautiful specimens of fossil plants, but so crushed and intermingled together that it is difficult to assign them to any known species of the fossil flora. There is sufficient reason for believing that the clay has been produced from the disintegration of the sandstone rock of the millstone grit series of our western moorlands, and that in the progress of the denudation of our moorland valley, it has been swept down from higher ground, and deposed on its present site, along with fragments torn from the rocks, and large boulders of "Ganister" from the parent rock in the Marsden Valley, and far away from their present resting place. In our field walls these large irregular boulders, hard as iron, and indestructible as granite, are often seen; they can be referred to no neighbouring quarry, and yet, perhaps, there are but few that think of the place from which they came, or of the velocity of the mighty wave of water that must have brought them here!

There is a rock of some thickness of argillaceous sandstone of smooth plain surface, which caps the high ridges of the hills to the eastward of this place; under it, alternating with beds of shale and banks of sandstone, lie the best seams of coal of the Yorkshire series. These beds of rich bituminous coal lie conformably with the rock that covers them, and therefore "crop" out, as the miners say, on the western sides of the hills, and disappear. The rock is found to take an irregular course from Derbyshire, Sheffield, Penistone, Whittey Hall, Grange Moor, and neighbouring hills, Elland Edge, and Clayton Heights. Passing Idle and Chapel Allerton, it returns parallel with the river Aire, to the magnesian limestone.

The town of Huddersfield is chiefly supplied with coal from Grange Moor and Flockton, a few miles distant; for the more valuable beds run out and disappear on the western side of the Whittey Hall and Grange Moor ridge of hills. We have, however, several lower beds of the series in the neighbourhood, but of less thickness and value than those of the upper part of the series. The different layers of coals appear to be characterised by a somewhat distinct flora. The upper beds, more particularly, contain beautiful specimens of tropical plants, calamites, lepidodendrons, etc.; and from the lower beds, worked in Grimescar wood, some of the finest specimens of ammonites have been seen, converted into iron pyrites, and being in appearance like gold. One of the lower beds of coal is worked at Steel Common, and fine fossil specimens of organic remains are frequently discovered. These lower beds, rising towards the moor lands to the west of Huddersfield and Halifax, soon run out and disappear almost entirely. The millstone grit rock, with its series of sandstone rocks and shales, appear and cover the moorlands through a large district of country. In this rock coal seldom appears, except in thin bands, which are supposed to have no connection with the general coal field. The chief member of the millstone grit series is a conglomerate rock, and forms the crags of our chief moorland eminences. It consists of fine large pebbles of quartz, water worn, and cemented within a mass of granular silicious particles, by the intervention of oxide of iron. We have specimens of it at Mytholm Bridge, Netherton, Quarmby, Halifax, and Harrogate. But its chief accompaniment is one of the finest siliceous grit stones containing magnesia; witness the beautiful—almost palatial—structures now rising in Huddersfield and Dewsbury.

This beautiful freestone is quarried at Marsh and Quarmby Edge, above this town. It is imperishable, and thoroughly impervious to moisture; and will ultimately render Huddersfield one of the finest towns in the kingdom. We must not forget to notice another rock, higher in the series; the fine slates and flagstones of Elland Edge, which have obtained, and most justly so, a wide reputation.

Water Supply. The qualities of the indispensable agent, water, must necessarily partake of the nature of the inferior beds or strata through which it has to percolate; and its purity will for the most part be in the indirect ratio of their solubility, with the exception of certain substances derived from another source, in the following manner. Rain water, descending upon and entering the ground, contains a notable quantity of carbonic acid and ammonia. The ammonia will be retained by the surface soil as a manurial agent; the freed carbonic acid will combine with the carbonate of lime and render it soluble, as also with the magnesia and protoxide of iron; part of these salts in solution may be required in vegetation, the remainder may be rejected as useless, and pass away in the water to the inferior strata.

But the chlorides of calcium and sodium are also always present in soils under cultivation. They are derived from the manure applied to the land for that purpose; and every particle of the manurial elements is firmly retained in the soil, while the chlorides for the most part are rejected, and not being necessary to vegetation, are likewise by descending rain removed from the soil and carried down to the inferior strata, and there accumulating, this water forms springs and streams for the future use of man. Hence it is that we find the purest spring water never entirely free from these mineral impregnations, as well as other ingredients, dissolved from the different strata it has had to traverse in its passage downwards. The quantities of these ingredients vary greatly: to the westward of Huddersfield the rocks are nearly insoluble; and, as our water is collected over a large area of the millstone grit series, it contains few ingredients except those, as before described, which must of necessity be present. The reservoirs for supplying the town are situated at a high level near Longwood; and the supply is very considerable from the natural springs which gush out from beneath the millstone grit rock. To increase that supply, several artesian wells have been bored to a considerable depth, which probably has not improved the purity of the water. This Longwood water was analysed some time ago by Mr. Marriott. The quantity of solid matter was inconsiderable, consisting principally of carbonate and sulphate of lime, and a trace of oxide of iron. The water of the Royleshead springs contained 2½ grains of solid matter in the gallon; of Bunney Clough, 1¾ grains; and of Cloughead 6½ grains. The latter contains some alumina; and all the samples contain a trace of chlorides and organic matter.

The supply of this excellent water to the township, and a few hundred tenements beyond, has been since last October 700,000 gallons per day. The average daily supply, throughout the year, is underrated at 450,000 gallons; which, distributed amongst 6,200 tenements, is in the proportion of 72 gallons to each, or between 13 and 14 gallons to each individual. This ample supply could very easily be enlarged, should the necessities of the town require it; for, at the present time, the waste during periods of overflux would be amply sufficient to fill another reservoir.

But to the south and east of this town the case is different. The descending rain has to pass through almost innumerable beds of shale (particularly to the eastward) bands of stonebind, soluble partly in acid, nodules of iron, and iron pyrites; and, on making its appearance as spring water, it is found in many cases of a very indifferent quality.

There is a very curious circumstance in connexion with the artesian wells of this neighbourhood. On penetrating beneath the fireclay and micaceous sandstone, a strongly alkaline water is obtained. In the valley to the south of Almondbury, at Rushfield, 375 feet above the sea level, this water was obtained at a distance of fifty-five yards from the surface ; on penetrating the micaceous sandstone, this water rose to a height of forty-eight yards. At a distance of one hundred and fifty yards from the above-mentioned well, another was sunk, and this water was reached eighteen yards from the surface. On the south side of Castle Hill, in the same valley, 600 feet above the sea level, the same strongly alkaline water was reached at a depth of twenty-eight yards ; it rose to within six yards of the surface. The Messrs. Brooke, of Armitage Bridge, and Mr. Atkinson, of Bradley Mills, have likewise bored, and found the same description of water, which has been analysed by Mr. Jarmaine, of Almondbury, and found to contain the carbonates of iron, lime and magnesia, silicate of soda, sulphate of soda, chloride of sodium, carbonate of manganese, and carbonate of soda, the latter in very considerable quantity.

The strong alkalinity appears to be due to the presence of the carbonate of soda. It is difficult to conclude otherwise than that these remarkable waters have a common origin. The places where they are found lie several miles apart, and at very different levels ; and if we admit that there is a natron lake in the bowels of the earth, whose waters exist under great pressure, still there are some circumstances in the case which appear difficult to explain.

The strata pierced through at Rushfield were as follows :—

| ft. | | yrds. | ft. | in. | |
|---|---|---|---|---|---|
| 1 | Alluvial soil ........ | 0 | 1 | 5 | Galliard—very hard. |
| 3 | Alluvial clay ........ | 5 | 0 | 7 | Stone bind. |
| 15 | Brown shale ........ | 20 | 1 | 6 | Black shale. |
| 21 | Blue shale .......... | 1 | 2 | 0 | Fire clay. |
| 35 | Black shale ........ | | | | |
| 4 | Stone bind .......... | 1 | 2 | 2 | Micaceous sandstone. |
| | | 55 | 2 | 8 | |

The river Colne washes the southern and eastern skirts of the town, and flows northward to the Calder ; it has suffered greatly from the intrusion of coal-pit water from the neighbourhood of Honley and other places, and is now loaded with sulphate of iron and other impurities. The proximity of a river should be conducive to the health and well being of a town's population, affording the opportunity for bathing, swimming, boating, and other pastimes of a pleasurable and salutary character, besides affording facilities for proper drainage.

But neither do our canal nor river serve any of these purposes, excepting the latter ; the streams are so dingy and dirty that they are unfitted for purposes of cleanliness ; instead of being the favourite promenade, the pedestrian who values his health avoids their banks, on account of their noxious exhalations, especially in the summer. The fish (their natural tenants) have departed, and their place is supplied by water rats, and those classes of small insects which defy, and seem to fatten on, putrescence.

INCREASE OF POPULATION. The population of Huddersfield has more than quadrupled itself during the last half century; in 1801 it numbered 7,268 persons, in 1851, 30,880. The average rate of increase of the whole British population is 1.3 per cent. annually; of manufacturing towns in Great Britain, 2.38 per cent.; whereas the increase of Huddersfield is considerably above 6 per cent. At the census in 1851, there were 5,739 inhabited houses, shewing an average of 5½ persons to each house in Huddersfield; the average of the whole of Britain was 5.7.

PAUPERISM. The amount of pauperism in Huddersfield is considerably below that shewn by an average of England and Wales. At the time of the census, 126,488 paupers were inmates of workhouses in England and Wales, being in the proportion of one indoor pauper to every 142 inhabitants, or seven in every 1000. In the half year ending September 29th, 1856, the total number belonging to this township receiving in-door relief was 211, being one in every 146 inhabitants, or seven in the 1000, not for one particular day, but for the whole half year. The total number receiving out-door relief pertaining to the township during the same period was 1,912; the number of lunatics chargeable to the township was 25, of which eight were in workhouses, and 17 either in asylums or receiving out relief. There are five workhouses belonging to the Huddersfield union, one of which is situated within the township. The number of paupers contained in this Huddersfield poor-house, on the average of 1854-55, was 330 per year; amongst these there were about 35 or 36 lunatics or idiots. The deaths in this institution average 45 in the year, or 14 per cent. of all admitted; whilst, taking an average of ten London workhouses, the mortality ranges as high as 20 per cent. Of the sexes, the males considerably predominate; and amongst the causes which attract to the workhouse, we find intemperance to be the most fertile in them, and prostitution in the female. This poor-house is a very inconvenient and badly-arranged building. Institutions like workhouses, where all the occupants are under direct control, should be model institutions; the inmates should especially be carefully classified; but in this one we find prostitutes mixing with the virtuous poor, and even with the children, and no distinctions between the idiots and the sane, the youthful and the aged. The sick wards and appurtenances are miserably contrived, and as an asylum for idiots or lunatics it is utterly unfitted; indeed, the proper places for these poor creatures are undoubtedly our County Lunatic Asylums, for in no poor-house is there the requisite organisation to admit of their receiving that attention which ought to be paid to mental diseases. If some of the workhouses of this union were united in a central and well-appointed building, and furnished with a thoroughly well-organised staff, I believe the comfort of the inmates would be materially increased, the claimants for admission uninjured, and the ratepayers in the long run relieved.

A suggestion has been made which seems deserving of a trial, viz., that amongst the females receiving relief, an attempt should be made to qualify some of them for the office of nurses to the sick poor who might require them, or those in better circumstances able to remunerate. No doubt many lives are lost among the poorer classes, and families for years made chargeable to the parish, for want of proper nursing in a serious illness; nor does it seem improbable that there may be many females now depending on the parish, who would be able to achieve their independence, and become useful members of society, if encouraged and stimulated by opportunity, discipline, and example.

(*To be continued.*)

---

